# Learning Through Reflection in Nursing Education: Perspectives of Nursing Students

**DOI:** 10.1177/23779608251407794

**Published:** 2026-01-29

**Authors:** Katrine Staats, Marita Nordhaug, Elin Thove Willassen, Inga-Linn N. Hansen

**Affiliations:** Department of Nursing and Health Promotion, Faculty of Health Science, 158921 OsloMet—Oslo Metropolitan University, Kjeller, Norway

**Keywords:** nursing students, reflection, education, learning, qualitative research, training

## Abstract

**Introduction:**

As healthcare evolves due to demographic changes and new technologies, there is a pressing need for nursing education to emphasize reflective practices. Despite its importance, students often struggle with integrating reflection into their learning due to inconsistent guidance and a lack of structured support.

**Objective:**

This study explored nursing students’ experiences and understanding of reflection, and how they engaged with it as a skill.

**Method:**

A qualitative exploratory design was employed, involving 22 1st- and 3rd-year nursing students. Data was collected through in-depth and focus group interviews, allowing for rich data collection through both interaction and individual insights. The data were analyzed using a six-step thematic process. The reporting followed the COREQ guidelines.

**Results:**

The analysis revealed two main themes, namely factors that support the reflection process and challenges of the reflection process with four subthemes: understanding the concept and purpose of reflection, educational structures and facilitation, lack of tools and structure in education, and power imbalances in relationships. Both 1st- and 3rd-year students found the concept of reflection broad and vague; however, 3rd-year students emphasized its practical applications. Both groups stressed the need for clear guidance and peer learning models to foster a supportive environment for reflection.

**Conclusion:**

This study identifies a gap between the ideal and actual experiences of reflection among nursing students. Both 1st- and 3rd-year students struggled to understand and apply reflection due to limited guidance. Education programs should introduce structured reflection training throughout the coursework, including consistent support from educators and supervisors. Addressing power imbalances could help establish a safe learning environment, enabling students to analyze their experiences both retrospectively and in real time. The findings offer insights that can enhance nursing education globally and contribute to the international knowledge base.

## Introduction

As healthcare services undergo rapid changes due to shifting demographics and the integration of new technologies, there is a pressing need for forward-thinking and reflective nursing education (International Council of Nurses, 2021). Nursing students are expected to address human needs, promote health, prevent and treat disease, alleviate suffering, and ensure a dignified death (International Council of Nurses, 2024; Regulation on National Guidelines for Nursing Education, 2019). To achieve these overall objectives, nursing students are expected to master the capacity for reflection (Helberget et al., 2020), as the concept of reflection is implemented in various learning outcomes (Regulation on National Guidelines for Nursing Education, 2019). For example, one states that the candidate “can reflect on and address ethical issues, as well as adjust their own practice in their service delivery” (Regulation on National Guidelines for Nursing Education, 2019, §8a). This learning outcome presupposes the *capacity* to reflect *on* a certain issue. Therefore, enhancing the reflective skills of nursing students is crucial (Meld. St. 38 [2020–2021]; Scheel et al., 2021). Nursing students’ ability to think critically and reflect increases when these skills are integrated into the university's program plan (Khalil & Abou Hashish, 2022; Pangh et al., 2019), which is crucial for understanding care and delivering effective healthcare services (Jaastad et al., 2022). However, there is a great deal of inconsistency in the use of reflective approaches and tools (Dahl & Alvsvåg, 2015; Scheel et al., 2021). This is related to several factors, such as teachers’ lack of knowledge of how to guide reflection, insufficient time allocated for guidance, and the use of many tools without understanding their purpose. Studies also indicate that nursing students often struggle to apply self-reflection, highlighting a pressing need for strategies that support reflective practice (Mlinar Reljić et al., 2019; Patel & Metersky, 2022).

## Review of Literature

Learning through reflection has long been a focus of research. Hallett (1997) and Smith (1998) emphasized that reflective learning strengthens both professional development and students’ capacity for emotional self-reflection. According to Bie (2020), reflection involves both practical skills and nurses’ critical thinking about various phenomena. It is a conscious act in which nurses critically examine their interpretations of the world. This aligns with American philosopher Donald Schön's two-part model: *Reflection-on-Action* and *Reflection-in-Action* (Schön, 1987, 2001). Reflection-on-Action involves reflecting after an event to understand what occurred, why it happened, and what can be done differently in the future. Schön (1987, 2001) also introduced Reflection-in-Action, which occurs during the event itself. According to Schön (1987, 2001), reflective practice fosters new ways of thinking by discovering insights both in real time and retrospectively.

Research emphasizes the importance of preparing nursing students for practice by incorporating reflection as an integral part of their education (Uswahzulhasanah & Arofiati, 2021; Vabo et al., 2021). Ideally, nursing students should be given opportunities to develop their reflective skills throughout their education to better integrate theory with practice. Reflection tools and models can support this process (Phenwan, 2023). Several international studies have presented reflection models and strategies used in nursing education (Bass et al., 2020; Boyd, 2023; Ooi et al., 2021). In Norway, two examples are the Centre for Medical Ethics (CMEs) dialogue model (Jakobsen & Sunde Maehre, 2023; Magelssen et al., 2016), and the Norwegian Nurses Association’s (NNA) five-step model (2015). The CME's dialog model helps nurses and students reflect on ethical dilemmas through structured discussions, integrating ethical theory with practical challenges to evaluate perspectives and make informed decisions. Similarly, the NNA's five-step model guides students in identifying dilemmas, analyzing situations, exploring actions, and reflecting on outcomes. Both models help nurses and nursing students reflect on ethical dilemmas in clinical situations. Additionally, research shows that both written and group reflection benefit learning (Monbec et al., 2021; Smit & Tremethick, 2017; Timpani et al., 2022; Zhang et al., 2023). However, few models or studies clearly define what reflection means for nursing students. Furthermore, previous studies have emphasized the need for more research to better understand what supports meaningful reflection processes for both teachers and students (Helberget et al., 2021; Jaastad et al., 2022). In light of this, the aim of the study is to explore nursing students’ experiences and understanding of reflection as both a means and a goal in their educational pathway. The purpose is to contribute knowledge about nursing students’ perspectives on reflection in education, enhance their reflection skills, and support the development of learning activities aligned with program plans and national regulations.

The research questions are as follows:

1. How do nursing students understand the concept of reflection within their educational context?
2. How do nursing students practically engage with reflection as a skill?

## Method

### Design

A qualitative, exploratory design was employed to study a concept about which little is known, focusing on the experiences of those involved (Creswell & Poth, 2018). The COREQ guidelines (Tong et al., 2007) were followed to promote comprehensive reporting of the study. Data was collected through in-depth and focus group interviews. In the focus group interviews, participant interaction and collective discussion were emphasized, allowing further exploration of emerging topics in subsequent in-depth interviews (Halkier, 2016; Kvale & Brinkmann, 2015). This dual-method approach enriched the dataset, treating both methods as equally valuable sources for addressing the study aim. The data were analyzed thematically using Braun and Clarke's six-stage inductive process (Braun & Clarke, 2022).

### Recruitment

The last author contacted the heads of nursing departments at two Norwegian universities. Both universities expressed interest in participating, however, one university ultimately decided not to proceed due to competing commitments and resource limitations at the time of recruitment. Digital information letters were sent to students via program directors, and the study was presented verbally during lectures. Despite a thorough recruitment plan (Negrin et al., 2022), challenges were encountered, including limited initial interest and scheduling conflicts among potential participants. To boost participation, a modest gift card was offered, a method recommended to improve response rates (Abdelazeem et al., 2023). From December 2023 to April 2024, 22 students consented to participate. Participants were purposively recruited to be in their 1st or 3rd year of the bachelor's in nursing program. Although students were grouped by year of study, it was acknowledged that their individual reflective competence could also have been shaped by personal circumstances and experiences. This approach was chosen to explore nursing students’ experiential foundations, expecting these to reflect developmental differences between the 1st and 3rd years.

### Data Collection

Data was collected through focus group discussions and individual interviews. Four focus group interviews were conducted with 18 students, divided into two groups of 1st-year students and two groups of 3rd-year students. The focus group discussion emphasized topics related to students’ understanding of reflection and its application in practice (Krueger & Casey, 2015). While larger groups were initially planned, challenges were encountered during the recruitment process. Some students who initially consented to participate withdrew at the last minute due to unforeseen circumstances. This resulted in one of the 1st-year focus groups consisting of only three participants. Given the logistical constraints and the timeline of the study, the decision to proceed with the smaller group size was made to ensure that all perspectives could still be captured. Although this group size deviated from the typical recommended minimum for focus group discussions (Krueger & Casey, 2015), the decision was justified to accommodate the challenges encountered during data collection, while still ensuring a diversity of insights and experiences. Splitting the 3rd-year students into two groups allowed for a more in-depth exploration of their reflections, as their advanced level of training often led to more detailed and nuanced discussions. The last author moderated all focus groups, with a coauthor serving as comoderator in each session. No one else was present besides the researchers and participants. The comoderator's responsibilities included taking detailed notes, observing group dynamics, and ensuring that key topics were addressed as planned.

A total of four in-depth interviews with nursing students were also conducted. These interviews deepened the understanding of how students comprehended and applied reflection in practice (Kvale & Brinkmann, 2015). The first author conducted three interviews; the last author conducted one. The sample size was determined based on the principle of data saturation (Creswell & Poth, 2018), and interviews were conducted until no new themes or significant insight emerged. The decision to stop after four participants was based on the homogeneity of the sample, as all participants were nursing students from the same institution, and by the focused scope of the research questions, which allowed for saturation to be achieved with fewer participants. Additionally, the richness and depth of the data collected provided sufficient insight to address the study's aim. All interviews took place at the institute where the students were affiliated. The venue and timing of the interviews were determined in collaboration with the participants to ensure convenience and flexibility. A quiet, private meeting room within the institution was designated for the interviews, providing a neutral and comfortable environment that minimized distractions and ensured confidentiality. To maintain flexibility and adaptability, the same semistructured interview guide was used for all sessions, tailored to individuals or groups. This approach allowed for consistency across sessions while enabling follow-up questions to explore emerging themes in greater depth. The interview guide was tested in a pilot interview with a small group of colleagues, who were experienced nurses and educators with expertise in nursing education, to refine the questions and ensure clarity. Data from the pilot testing was not included in the main analysis. Examples of questions included: “How do you understand the concept of reflection?” and “Can you describe a learning situation in which you had to reflect on something verbally or in writing?,” All interviews were audio-recorded and transcribed verbatim. Participants were informed about the recording process in advance and provided written consent, ensuring ethical compliance and transparency. Focus group interviews lasted between 39 and 60 min, and in-depth interviews ranged from 51 to 58 min.

### Rigor

To ensure the trustworthiness of this study, the criteria outlined by Lincoln and Guba (1986, 1994): credibility, dependability, confirmability, transferability and authenticity were adhered to. Credibility and dependability were ensured through a thorough reflection on the researchers’ preunderstandings, as well as maintaining transparency throughout the study's design, data collection, and analysis processes. Confirmability was strengthened by focusing on capturing the nursing students shared experiences, while continuously reflecting on and addressing the researchers’ preconceptions. Disconfirming evidence within the data was also sought to minimize confirmation bias. To enhance transferability, sufficient data was collected to create a rich and thick description of the students’ experiences, ensuring that the findings would be relevant in similar contexts (Lincoln & Guba, 1986). Lastly, authenticity was ensured by highlighting the students’ voices and presenting their experiences as authentically as possible, in alignment with their own experiences (Lincoln & Guba, 1994). These measures collectively ensured the rigor of the research process and findings.

### Data Analysis

The focus group and in-depth interview data were analyzed using Braun and Clarke's six-step reflexive thematic analysis (Braun & Clarke, 2022). Initially, the transcripts were read individually to identify and highlight students’ experiences with reflection. Next, the meaning units were categorized into codes to identify similarities and differences in the text. In the third step, the transcripts were reviewed again to find examples of how each theme or category appeared in the data. These examples were then grouped into appropriate themes to identify patterns in students’ experiences with reflection. In the fourth step, the themes were refined and reviewed to ensure clarity, coherence and distinction, with overlapping themes combined and irrelevant data excluded. In the fifth step, the finalized themes were defined and named to accurately present their core meanings and relevance to the study aim. Finally, in the sixth step, the text of the article was produced, and representative quotes were selected to illustrate each theme and subtheme in a suitable order (Table 1). Throughout this process, reflexivity was prioritized by critically reflecting on the roles of the researchers and their preconceptions, acknowledging how professional backgrounds and personal biases could influence the analysis. To address this, ongoing discussions were held during collaborative analysis meetings, where interpretations of the data were presented and debated. This iterative dialogue challenged potential biases and ensured that the themes were grounded in the participants’ narratives rather than the researchers’ assumptions. This collaborative process enhanced the credibility of the analysis and supported the maintenance of a reflexive stance throughout the study.

### Ethical Considerations

The research was conducted in accordance with ethical practices outlined in the Helsinki Declaration (World Medical Association, 1964/2024). Special attention was given to the ethical obligation to prevent negative experiences for participants. “Negative experiences,” referred to any discomfort, hesitation, or feelings of vulnerability that participants might encounter during the interviews or focus group discussions. When students showed discomfort, reluctance to share, or a tendency to give “correct” answers—responses they believed were expected—they were met with understanding. Information about confidentiality and the voluntary nature of participation was reiterated, and it was emphasized that there were no consequences for withdrawing from the study at any time before publication. Participants were assured that they could withdraw without any explanation or repercussions. Anonymity was ensured by removing all identifying information during transcription, and no names of personal details were included in the data analysis or reporting. Participants received detailed information about the study, both orally and in writing, and signed the informed consent form before taking part in the study. Data were collected and stored in Nettskjema; a tool developed for secure data storage in compliance with the Personal Data Act (2018). All data will be deleted upon completion of the research project. The project is registered with the Norwegian Agency for Shared Services in Education and Research (Sikt; ref. 644484).

## Results

A total of 22 participants (14 females and eight males) took part in the study, which included four focus group discussions and four individual in-depth interviews. The participants were evenly distributed between 1st year (*n* = 11) and 3rd year (*n* = 11) nursing students (Table 2).

**Table 1.** Themes and Subthemes Identified in the Data.

Theme	Subtheme	Patterns/meaning units	Data extract
Factors that support the reflection process	Understanding the concept and purpose of reflection	Uncertainty about purpose	“I didn’t really understand what reflection was supposed to achieve.”
	Educational structures and facilitation	Role of educational support	“It helped when the teacher gave us a structure to follow.”
Challenges of the reflection process	Lack of tools and structure in education	Absence of practical support	“We were told to reflect but not given any tools or examples.”
	Power imbalance in relationships	Relational barriers to reflection	“I didn’t feel comfortable sharing openly with my supervisor.”

**Table 2.** Overview of the Study Participants.

	Gender	Age	Year of Study
Focus group interview 1:	F (1), M (2)	Range 21–27 y (mean 23)	1st year
Focus group interview 2:	F (5), M (1)	Range 20–32 y (mean 24)	1st year
Focus group interview 3:	F (4), M (1)	Range 22–35 y (mean, 27)	3rd year
Focus group interview 4:	F (2), M (2)	Range 27–39 y (mean, 34)	3rd year
In-depth interview 1:	F	38	1st year
In-depth interview 2:	M	20	1st year
In-depth interview 3:	F	42	3rd year
In-depth interview 4:	M	24	3rd year

The analysis revealed two primary themes: factors that support the reflection process and challenges of the reflection process. Each theme had two supporting subthemes namely understanding the concept and purpose of reflection, educational structures and facilitation, lack of tools and structure in education, and power imbalances in relationships respectively. Table 2 provides an overview of the identified themes and subthemes. These themes and their respective subthemes are subsequently presented.

## Theme 1: Factors That Support the Reflection Process

This theme describes the elements that promote and facilitate nursing students’ ability to engage in reflection during their education. Specifically, it highlights the importance of understanding what reflection is and its purpose, as well as the role of educational structures and facilitation in fostering reflective skills. The theme is supported by two subthemes: *understanding the concept and purpose of reflection* and *educational structures and facilitation.*

### Understanding the Concept and Purpose of Reflection

First-year students described reflection as a multifaceted, personal process. They associate it with exploring ideas and viewing them from different perspectives. They used terms like “thinking,” “being critical of something,” “delving deeper,” and “a process.” For many, these ideas blended together, suggesting not just thinking but also absorbing impressions and considering issues from multiple angles. However, most of the 1st-year students found the concept of reflection broad and vague. Nevertheless, they noted using reflection unconsciously, often triggered by questions from teachers that helped initiate the process. A 1st-year student noted that reflection was a natural part of her education, often occurring informally with peers:

At first, I think … what do you mean by reflection? But then I actually think that reflection is always about how you think more about something or have some thoughts about something you have read, or perhaps about yourself. I haven't noticed that it is part of the curriculum, to be honest. I believe I have reflected a lot, and maybe also together with others, without really noticing it. (In-depth 1st-year, female)

Third-year nursing students focused more on the practical applications of reflection, discussing its “why” and “how” rather than its fundamental meaning. Several students still described reflection as a vague, overwhelming concept—one stating it is “so broad that it becomes narrow” (Focus group 3rd-year, female). For most 3rd-year students, reflection has become an integrated part of their practice, even if not explicitly named. They noted that reflection requires maturity developed through experience, in contrast to their 1st-year perspectives. One student shared this experience from his placement in home care services:

We had a challenging situation with blood sugar levels that didn't make sense. Two years ago, I would have thought, yes, high blood sugar, what more can you do? But now, within the next half hour, I considered cardiovascular diseases, liver diseases. I had a very broad perspective on it and was discussing it with myself while driving back to the office. So, I was reflecting in the car, something I wouldn't have done two years ago. (Focus group 3rd-year, male)

This example shows how a 3rd-year student indirectly understood the purpose of reflection, using it to navigate the clinical situation and sharpen his critical thinking.

### Educational Structures and Facilitation

To support reflection, both 1st- and 3rd-year students emphasized that the institution should actively provide guidance and structured templates. Few students were familiar with reflection tools, and several noted that understanding the concept of reflection is essential to use such tools effectively. Particularly, for 1st-year students coming directly from high school, taking on reflection tasks felt like a major shift in responsibility, especially when assigned reflection tasks by their teachers. Despite finding the concept of reflection challenging to grasp, they recognized the importance of engaging in reflective practices. As such, they expressed a need for courses or a precise “recipe” to guide them through a reflection process, incorporating learning outcomes and prompting questions. One student highlighted the institution's responsibility to teach both what reflection is and how to apply it in practice:

I think that a form of seminar in reflection and critical thinking starting in the first year, where you begin with basic reflection, would be beneficial. In the second year, you could take it further, and in the third year, we could focus on independent reflection and independent thinking, creating a progression that matches the learning or educational journey. Instead of going from knowing nothing to suddenly being on your own. (Focus group, 3rd-year, male)

Additionally, oral guidance from supervisors on what reflection entails and how to engage in it effectively was considered essential. The initiation of reflection often depended on the supervisor's experience and attitude, as they were seen as a “driving force” in the process. Students noted that supervisors should structure the daily schedule to allow time for reflection, though this varied by practice environment. For example, home care services offered more opportunities for reflection than hospital wards, while psychiatric settings explicitly set aside time for it. Furthermore, peer learning also encouraged reflection among 3rd-year students. Pairing students in the same ward—“tospann modellen” in Norwegian—made reflection feel more natural and less intimidating. Students felt safer asking a fellow student questions than a supervisor, whose role was to assess them. This peer interaction fostered a more open and supportive environment for reflection:

If the school were to incorporate reflection to a greater extent, they should definitely focus more on “tospann modellen.” I have never gained as much from reflecting as I did when working in a pair. We could work more closely together, making it easier to ask questions like “Why do you do what you do, and why do I do what I do?” There is an incredible amount of learning in that. (Focus group 3rd-year, female)

Third-year students in both focus groups emphasized that supervisor guidance and peer learning through “tospann modellen” were crucial for developing reflective skills. They agreed that structured support should be provided from the 1st year to help them navigate the reflection process.

## Theme 2: Challenges of the Reflection Process

This theme highlights the barriers that hinder nursing students from effectively engaging in the process of reflection during their education. It emphasizes the lack of tools and structured support for reflection and the challenges posed by power imbalances in relationships with teachers and supervisors. These obstacles often left students feeling uncertain, unsupported, and unable to reflect meaningfully. The theme is supported by two subthemes: *lack of tools and structure in education* and *power imbalance in relationships*.

### Lack of Tools and Structure in Education

Students identified the lack of clear frameworks and tools as a major barrier to reflection. Students generally reported receiving insufficient information about reflection during their 1st year. They noted a lack of training; they were told to reflect but not taught how to do it as a skill. In their 1st year, students completed group exams that ended with a question asking each member to reflect on their contributions. This practice was deemed impractical by the participants:

You couldn't be honest in the group work about how you reflected on yourself, on your effort, and what your contribution was, because everyone could see it … You weren't really honest with yourself, because you couldn't see the others’ feelings, so you just wrote nicely and submitted it as a group. (Focus group 1st-year, female)

Students also highlighted the lack of structured support for reflection from their teachers. Students in both the 1st and 3rd year reported inconsistent expectations from teachers and supervisors in practice. Some teachers wanted reflections to be grounded in theory, while others encouraged more personal insights. This inconsistency created confusion:

I feel it varies a lot depending on the teacher. Some have said we should base our reflections on the curriculum, but then it feels more like repeating the curriculum. Others have asked ‘Why do you do that?’ which I feel creates reflection just by asking. (Focus group 3rd-year, female)

Additionally, according to the participants, unclear and complex learning outcomes made it challenging to engage in reflection. Many students found the learning outcomes to be vague and difficult to interpret, especially those including reflection. They struggled to understand what was expected of them:

When I read the learning outcome, I just stopped when I saw the word and thought to myself, what do they want me to talk about? It's like, yeah, but where should I start? What do you want me to include? (Focus group 1st-year, female)

Even without clear frameworks and guidance on how to use the concept of reflection, the 3rd-year students in this study demonstrated a growth in their reflective skills. However, they found it demanding to understand learning outcomes that contain the concept of reflection and connect theoretical knowledge into practice. They emphasized the importance of practicing supervisors understanding and supporting the learning outcomes. Without this support, students felt overwhelmed and isolated, making it difficult to reflect effectively. One student highlighted:

I think the most important thing is that the supervisor is committed to the learning outcomes. They must bring it up and ensure that the student reflects. When the supervisor sees us running around with no time, I think it is their responsibility to say stop! We need to sit down and reflect. (Focus group 3rd-year, female)

Third-year students particularly noted a lack of culture of reflection in many settings. They expressed that many practice placements did not prioritize or facilitate reflective practices. This absence of a reflective culture made it challenging for students to engage in meaningful reflection, as they often lacked the necessary time and support from their supervisors.

### Power Imbalance in Relations

The students in this study highlighted a power imbalance in their relationships with teachers and supervisors, which they felt affected their ability to engage in meaningful reflection. Both 1st- and 3rd-year students reported that they often reflected not primarily for their own learning, but to meet the expectations of their teachers. They were more concerned about what the teacher was looking for and strove to deliver what they believed the teacher wanted. Third-year students, in particular, expressed hesitation in initiating reflection due to fears that their comments could be used against them. They felt they were at the mercy of their supervisors’ assessments and were worried that any perceived criticism might negatively impact their evaluations. As discussed in a focus group:

If I am in practice and see the supervisor doing something wrong, I keep my mouth shut. I don't want the supervisor to get a bad impression of me. … And then I reflect by myself. (Focus group 3rd-year, female)

I feel a bit that the supervisor knows everything. And you are kind of ‘under.’ But sometimes I think we know more because we are up to date. But there is a strong focus on the supervisor teaching you, and not the other way around. (Focus group 3rd-year, male)

The 1st-year students described some experiences with receiving abrupt responses when asking genuine questions due to hecticness. As such, they were afraid to invite to reflections, fearing negative consequences. This could threaten the power dynamics and lead to feelings of shame or fear of payback. A 3rd-year student raised the question whether the concept of reflection is useless when you only reflect to give the teacher what they want, using the teacher's preferred language or just make something up to reflect on, without engaging in self-reflection:

If the supervisor or teacher wants a reflection, you can just refer to something you did during the day. So, in a way, you feel a bit forced into reflection if the supervisors are very insistent. (In-depth 3rd-year, male)

The results show that both 1st-year and 3rd-year students experience a power imbalance between students and their supervisors, which hinders the reflection process. They often felt compelled to reflect not to support their own learning, but to satisfy their evaluators, leading to reflections that felt inauthentic. Furthermore, many students expressed a fear of negative consequences and felt that being critically reflective discouraged open and honest engagement in the reflection process.

## Discussion

Both 1st and 3rd-year students described the concept of reflection as complex. First-year students often described reflection as a vague and subjective concept, while 3rd-year students viewed it more practically, integrated into their clinical practice. However, 3rd-year students, similar to 1st-year students, still found reflection difficult to fully comprehend, both as a concept and as a tool. The results of this study highlight the need for a clear understanding of the concept and purpose of reflection to help students navigate the reflection process, as well as thorough guidance from teachers and an opportunity for peer learning. Conversely, several barriers hinder effective reflection, such as a lack of training and support from teachers and supervisors in practice. Additionally, complex learning outcomes and power imbalances in relation to teachers challenge students’ ability to reflect. These findings will be discussed in relation to previous research and Schön's twofold model of *Reflection-on-Action* and *Reflection-in-Action*. These perspectives will be integrated with the research questions, presenting two key themes in this discussion:

### From Struggle to Practice: Nursing Students’ Evolving Journey With Reflection

The 1st-year students in this study found reflection a challenging and broad concept, encompassing terms like thinking, being critical, delving deeper, and viewing issues from various perspectives. This applied to both written and oral reflections, with written reflection being particularly challenging during the 1st year of study. This aligns with a study by Bjerkvik and Hilli (2019), which states that most students reflect primarily at a descriptive level, thus showing limited reflective skills. Bjerkvik and Hilli (2019) claim that reflective writing is crucial for students’ personal development as professional nurses. It is therefore not surprising that 1st-year students find the reflection process particularly challenging. Nursing bachelor program descriptions also emphasize the importance of reflective capacity, expecting students to master reflection as a skill, with personal development arising from their own actions. One way to develop such skills and help students identify issues for reflection is through the use of reflective tools and models (Jakobsen & Sunde Maehre, 2023). However, as noted by the nursing students in this study, few were familiar with reflection tools, even though some were introduced during the 1st year. Students noted, however, that one must understand the concept of reflection to use such tools effectively. Additionally, this study found that 1st-year students struggled with the responsibility of developing their own understanding of reflection as a learning tool. Damsa and de Lange (2019) emphasize that this responsibility cannot be left to students alone; they need explicit support from teachers within their learning environment to learn how to reflect on learning situations. Moreover, reflection requires both personal and professional development over time, influenced by experiences in clinical placements and enhanced professional knowledge. Consequently, it may be beneficial to position learning outcomes related to reflection later in study programs, allowing these skills to develop more thoroughly and equitably.

The 3rd-year students emphasized a more practical application of reflection, focusing on its “why” and “how.” However, like the 1st-year students, 3rd-year students found the concept of reflection broad and overwhelming. They emphasized the school's support for reflective learning in clinical placements, preferring the “tospann model,” which pairs two students at the same placement to foster a safe, open, and supportive environment for reflection. A review by Koch et al. (2023) underscores the effectiveness of this practice model, identifying it as particularly successful because students benefit both psychologically and professionally from peer support. Students gain greater independence by sharing responsibility. A study by Zhang et al. (2023) adds to this discussion by showing how peer feedback enhances nursing students’ reflective abilities, which might also improve their clinical competence. From the supervisors’ perspective, students were more eager to learn and actively engaged in reflective discussions, both in pairs and with their mentors. Additionally, this approach was found to be time-saving and efficient for supervisors (Koch et al., 2023). Nevertheless, as found by Choi et al. (2021), peer learning will not impact the level of reflection without proper tutoring and a supportive educational environment. A recent Norwegian governmental report (Meld. St. 24 [2019–2020]) calls attention to the importance of organized and adapted reflective guidance across all levels of the health and care services. Therefore, to achieve these targets, it seems crucial to focus on facilitating and structuring resources to help students navigate the reflection process, starting early in their nursing education.

One way to understand the findings and contribute to the knowledge of how nursing students should navigate the reflection process is to view it through the lens of Schön's theoretical model: “Reflection-on-Action” (Schön, 1987). This model involves looking back to understand the outcomes of actions and thinking analytically about what happened. It allows for learning from experiences, why something happened, and how it could be done differently in the future (Schön, 2001). By adopting the “Reflection-on-Action” mindset, teachers and supervisors can guide students to retrospectively analyze their reflections, helping them identify which aspects of their writing were effective and which were not. A frequently used method for ethical reflection that can also be applied retrospectively is the CME's model (Jakobsen & Sunde Maehre, 2023). The CME's stepwise reflection model, integrating knowledge from various domains nursing, ethics, law, and the perspectives of involved parties, is a useful tool for nursing students’ oral and written reflections.

The perspective “Reflection-on-Action” can lead to a deeper understanding of students’ reflective practices and highlight areas for improvement in reflection as a tool for learning. Additionally, it might increase students’ awareness of the concept of reflection and provide them with opportunities to practice and become more familiar with it. Similarly, 3rd-year students emphasized the practical application of reflection and the importance of educational support in learning how to apply it. Using Reflection-on-Action, these students can review their experiences—for example, with the “tospann model”—and analyze how peer learning facilitated their reflective processes. Moreover, explicit support from teachers and clear explanations of what reflection is and how to use it are essential for effectively implementing Reflection-on-Action. Therefore, as stated by Schön (1987), Reflection-on-Action offers a valuable opportunity to integrate theory and practice, enabling students to identify patterns that might not have been evident in the heat of the moment.

### Reflection in the Moment: Barriers and Opportunities for Growth

In addition to seeing reflection as a learning goal that allows students to understand the outcome retrospectively (Reflection-on-Action), many nursing students explained how reflection occurred naturally in a process known as “Reflection-in-Action” (Schön, 1987). First-year students described how this could happen unconsciously and was often triggered by teachers’ questions that facilitated the reflection process. They also described it as a spontaneous process that occurred during dialogs with peers. This was particularly true for older students (25 and above), who articulated how this development helped them become reflective learners. Third-year students echoed this sentiment, noting that reflection requires a maturity developed over time—both in age and professional development—in contrast to their 1st-year experiences. Interestingly, these results can be understood in the context of life stages and cognitive development in young adults. According to Arnett et al. (2014), individuals aged 19 to 25 may lack fully developed cognitive maturity related to behavior and emotions, making it challenging for them to engage in reflection. Reflective regulation continues to develop into adulthood, with opportunities for reflection increasing with age (Ferschmann et al. 2021; Zelazo & Doebel, 2015). Therefore, this study can infer that the ability to reflect as a means is not fully developed at age 20—the average age of students—but is an ongoing process of self-understanding. This raises a critical question: How can it be expected that students possess reflection skills initially? As suggested by the informants themselves and supported by Pretorius and Ford (2016), a structured program of reflective training and guidance should be offered progressively over the 3 years of study to positively influence and support students’ reflective development.

Nevertheless, all types of programs presuppose a certain foreknowledge of how to reflect, as they are based on the idea that reflection fosters both active and dynamic learning (Boyd, 2023). Therefore, skilled and supportive teachers are essential to this process. However, both 1st- and 3rd-year students in this study experienced inconsistent expectations from teachers regarding reflection and how to apply it in practice. They also found the learning outcomes vague and difficult to interpret and particularly struggled to understand how to reflect. They stated that supervision is a prerequisite and described how they found it challenging to be in placements that lacked a culture of reflection. Moreover, research shows that over time, supervisors spend progressively less time reflecting in clinical practice with their nursing students (Shafakhah et al., 2016). Another issue is that the development of students’ reflection skills was hindered by fear of negative consequences, as some engaged in artificial reflection merely to satisfy the supervisor or teacher. Such a power imbalance may even contribute to misinformation or misguided reflection in the learning process. Similar findings are documented by Barbagallo (2021), who describes how students often experience fear and anxiety about being judged, which hinders their ability to engage in reflective personal growth. According to Andersen et al. (2018), nursing students sometimes even feel pressured to participate in reflective discussion, leading to a negative attitude toward reflective learning. All of these are factors that hinder nursing students from developing their reflective skills and becoming reflective nurses.

To optimize reflective learning, it may be fruitful to interpret the findings in light of Schön's second component of the theoretical model: Reflection-in-Action (Schön, 1992). This highlights a key connection between practice and theory—carrying out reflection as the situation unfolds, not in retrospect. This immediate reflection helps adjust decisions on the spot and requires a certain level of self-awareness and critical evaluation. This also depends on individual tacit knowledge—knowing-in-action (Schön, 1992)—which nursing students have developed over time (Elsayed et al., 2024). Findings indicate that nursing students are often prevented from engaging in these processes due to various barriers. For example, the lack of training and clear frameworks for reflection resonates with Schön's assertion that Reflection-in-Action is a skill without rules and procedures (Schön, 1992). This form of reflection requires clinical judgment, spontaneous behavior, and cognitively developed reflective skills. Therefore, for Reflection-in-Action to be effective, students need the space and support to engage with the “situation” (their experiences) and learn from it, as well as sufficient cognitive maturity (Arnett et al., 2014). Nevertheless, the lack of a reflective culture in placements and the power imbalance between students and supervisors often prevent them from practicing and refining their reflective skills in “real world settings.” Fear of negative evaluations stifles critical reflection and prevents students from engaging meaningfully with the situation. This power imbalance directly contradicts the principles of Reflection-in-Action, where open dialogue and feedback “on the spot” are crucial for learning and growth (Schön, 1992). In this study's findings, students who kept quiet about a supervisor's mistake exemplify this. They are prevented from engaging in honest reflection on their practical knowledge due to fear of repercussions. This highlights a significant gap between the ideal of Reflection-in-Action and the students’ actual experiences.

To become a truly reflective nurse, there must be a balance and dialogue between the two aspects of Schön's model of reflection. This means that to recognize phenomena, such as signs and symptoms associated with a particular disease, nursing students must be able to engage in Reflection-in-Action and have the opportunity to develop these skills. It is equally crucial for them to develop the ability to reflect on situations (Reflection-on-Action) as both a tool and a goal for learning. According to Schön, this means that Reflection-on-Action can lead to Reflection-in-Action. Importantly, Gjerberg et al. (2017) states that Reflection-on-Action may be useful in situations requiring Reflection-in-Action. According to Gjerberg et al., the interaction between these two forms of reflection is stimulated by reflecting on past events together with others. Therefore, fostering both forms of reflection is essential for the comprehensive development of reflective skills in nursing students.

### Strength and Limitations

The study has several strengths. Including both 1st- and 3rd-year students provides a breadth of experience. Conducting research within the researchers’ own institution presented certain advantages, such as familiarity with the program's curriculum and learning activities, which enabled more effective follow-up during interviews. Additionally, offering both focus group and individual interviews allowed students who preferred not to be in a group setting to share their experiences. Finally, the involvement of four authors in the analysis process was a significant strength, as this collaboration enhanced the objectivity and nuance of the analysis, ensuring a well-rounded interpretation of the data.

However, the study also has limitations. It only involved nursing students from one bachelor's program at a single university. While different programs may emphasize reflection differently in their curricula, potentially limiting external validity, Norwegian universities adhere to national and EU guidelines, ensuring standardized education (Universities Norway, 2016). A limitation of the study was the small size of one of the 1st-year focus groups, which consisted of only three participants. This was due to challenges in recruiting participants at the time of data collection, as several students who had initially consented to participate withdrew at the last minute. Despite this, the discussion with the three 1st-year students was rich and meaningful, providing valuable insights. In hindsight, this was acknowledged as a methodological limitation and recognized that combining all 1st-year students into one group could have been a better approach to align with standard focus group sizes (Halkier, 2016). Additionally, the recruitment method may have resulted in participation primarily from students who were more comfortable reflecting on and sharing their experiences, potentially limiting the diversity of perspectives and affecting internal validity. Another limitation was that students may have known the researchers, which could have influenced their openness. To mitigate this, interviewers were not involved in teaching the relevant courses or year units. Efforts were made to remain open to potential biases and maintain objectivity when engaging with the material.

### Implications for Nursing Education

The findings of this study underscore the critical need to rethink how reflection is integrated into nursing education. Reflection is not just a theoretical exercise but a skill that must be taught, supported, and practiced systematically. This has implications for both the design of nursing curricula and the broader educational strategies used to prepare students for the complexities of modern healthcare. Specifically:

- Reflection must be framed as a developmental process, evolving from foundational skills in the 1st year to advanced reflective practices in the 3rd year.
- Addressing power imbalances in student–supervisor relationships is critical to fostering a safe and supportive learning environment.
- Peer learning models, such as the “tospann model,” show promise in creating collaborative spaces where reflection can thrive.
- Educators and supervisors play a pivotal role in modeling and facilitating reflective practices, which require targeted training and resources.

By addressing these implications, nursing education can better prepare students to use reflection as a tool for critical thinking, self-awareness, and professional development.

## Conclusion

This study revealed a significant gap between the ideal of reflection and the actual experiences of nursing students. Both 1st- and 3rd-year Norwegian nursing students struggled to understand and apply the concept of reflection in their practice. Existing models of reflection offered limited guidance on how to practice and develop this essential skill. To enhance the development of reflective skills in nursing students, education programs should introduce structured reflection training throughout the coursework. Consistent support from educators and supervisors is essential, along with the adoption of peer learning models such as the “tospann model.” By addressing power imbalances between students and supervisors, a safe learning environment can be established, enabling students to analyze their experiences both retrospectively and in real time. Without these elements, reflection risks becoming a superficial exercise, failing to deliver the deep learning and self-awareness it promises. Implementing these measures will better prepare nursing students to navigate the complex and evolving healthcare landscape, contributing to the development of both personal and professional reflective capabilities. Though focused on Norway, the findings offer insights into the purpose of reflection in nursing education that can enhance programs globally and contribute to the international knowledge base.
